# Delayed Endothelial Progenitor Cell Therapy Promotes Bone Defect Repair in a Clinically Relevant Rat Model

**DOI:** 10.1155/2017/7923826

**Published:** 2017-03-30

**Authors:** Brent D. Bates, Charles Godbout, David J. Ramnaraign, Emil H. Schemitsch, Aaron Nauth

**Affiliations:** ^1^Keenan Research Centre for Biomedical Science, St. Michael's Hospital, University of Toronto, 209 Victoria Street, Toronto, ON, Canada M5B 1T8; ^2^Department of Surgery, London Health Sciences Centre, 339 Windermere Road, London, ON, Canada N6A 5A5; ^3^Department of Surgery, Division of Orthopaedics, St. Michael's Hospital, University of Toronto, 30 Bond Street, Toronto, ON, Canada M5B 1W8

## Abstract

The repair of segmental bone defects remains a significant challenge for orthopaedic surgeons. Endothelial progenitor cells (EPCs) have successfully promoted the repair of acute defects in animal models; however, the ability of EPCs to induce the repair of chronic nonhealing defects, such as those often encountered clinically, has not been investigated. Therefore, the purpose of this study was to investigate the ability of EPCs delivered in delayed fashion to induce the repair of nonhealing defects in a clinically relevant model. In order to simulate delayed treatment, 5 mm segmental defects in Fischer 344 rat femora were treated with bone marrow-derived EPCs on a Gelfoam scaffold at 3 weeks post creation of the defect. At ten weeks posttreatment, 100% of EPC-treated defects achieved union, whereas complete union was only achieved in 37.5% of defects treated with Gelfoam alone. Furthermore, significant increases in ultimate torque (*p* = 0.022) and torsional stiffness (*p* = 0.003) were found in EPC-treated defects compared to controls. Critically, no differences in outcomes were observed between acute and delayed EPC treatments. These results suggest that EPCs can enhance bone healing when applied in an acute or delayed fashion and that their use may represent a clinically translatable therapy for bone healing in humans.

## 1. Introduction

Despite recent advances in surgical techniques and implants, the repair of bone defects and nonunion secondary to trauma or infection remains a significant challenge. Fracture nonunion is a debilitating condition that substantially impacts health-related quality of life and creates a significant burden on healthcare systems [1–3]. In the tibia alone, open fractures develop nonunion in 23% of cases [3] and demonstrate increasing complication rates as fracture severity and bone defect size increase [4–6]. Autologous iliac crest bone grafting (AICBG) is the current gold standard of treatment for bone defects; however, bone grafting is associated with overall complication rates of 19% [7] and is further limited by the amount of bone available, donor site morbidity, and suboptimal healing outcomes [7, 8]. Other treatment modalities including vascularized autografts, cortical allografts, and the Ilizarov technique have also been utilized for the repair of bone defects [9]. However, similar to AICBG, each of these techniques have demonstrated high complication rates and substantial limitations in efficacy. A variety of tissue engineering substitutes, including osteoinductive molecules such as bone morphogenetic proteins (BMPs), osteoconductive scaffolds such as calcium phosphates, and osteoprogenitor cells such as mesenchymal stem cells (MSCs), have been investigated as potentially superior treatments for bone defects [10, 11]. However, to date these therapies have failed to translate into clinical practice. One potentially significant limitation of each of these therapies is their lack of angiogenic capacity and failure to address blood supply to the tissue-engineering construct [12].

Accordingly, angiogenic cell populations, such as EPCs, have recently been investigated, and our research group [13–16], as well as others [17–19], has successfully demonstrated the ability of EPCs to affect the repair of segmental bone defects in animal models when the cells are applied acutely to freshly created defects. However, the timing of intervention plays a significant role in the efficacy of both cellular and molecular therapies [20, 21] as a result of the differing inflammatory environments at different time points following trauma. Inflammatory cytokines released immediately after fracture, such as interleukin (IL)-1 and IL-6 [22, 23], have been shown to stimulate EPC proliferation, migration, adhesion, vascular endothelial growth factor (VEGF) expression, and tubulogenesis [24–26], suggesting that local inflammation at the fracture site has the potential to stimulate EPC-mediated repair of acute defects. However, in the clinical context of open fractures complicated by bone loss, bone grafting is most commonly delayed to minimize graft resorption, reduce infection risk, and allow soft tissue healing at the fracture site [27]. Furthermore, for fractures that require bone graft treatment for delayed union or nonunion, treatment would typically occur outside of the initial inflammatory window. Therefore, the environment in which bone grafting or the application of bone graft substitutes would typically occur in the clinical situation has not been well replicated in animal models of EPC therapy, which apply the cells to acutely created bone defects. The current study sought to address this by investigating EPC therapy in a more clinically relevant model of delayed treatment.

The primary objective of this study was to investigate the use of ex vivo expanded EPCs for the regeneration of bone defects in a clinically relevant model of delayed treatment using a chronic bone defect. In addition, we sought to compare delayed delivery of EPCs to EPCs delivered in an acute fashion to evaluate the effects of treatment timing on functional bone repair. We hypothesized that delayed treatment with ex vivo expanded EPCs surgically transplanted into chronic bone defects would enhance bone repair in a comparable fashion to acute treatment.

## 2. Methods

### 2.1. Experimental Design

Thirty-six male Fischer 344 syngeneic rats weighing 250–300 g underwent open surgery to create a 5 mm segmental defect in the mid-diaphysis of the right femur, which was then stabilized with a miniplate and screws. Animals were then randomly assigned to one of five groups: (1) delayed EPC group: delayed grafting at 3 weeks post bone defect creation with Gelfoam scaffold (Pfizer, New York City, NY) loaded with 1 × 10^6^ culture-expanded EPCs in cell culture medium (*n* = 8); (2) delayed control group: delayed grafting at 3 weeks post bone defect creation with Gelfoam control (no cells, soaked in cell culture medium only) (*n* = 8); (3) empty defect control group: no additional treatment at 3 weeks post bone defect creation (*n* = 8); (4) acute EPC group: immediate grafting of bone defect with Gelfoam scaffold loaded with 1 × 10^6^ culture-expanded EPCs in cell culture medium (*n* = 6); or (5) acute control group: immediate grafting with Gelfoam control (no cells, soaked in cell culture medium only) (*n* = 6). Animals receiving immediate treatment were sacrificed after 10 weeks. In the delayed treatment and empty defect control groups, defects were left empty for the initial 3-week delay, and animals were sacrificed 10 weeks thereafter. All animals were sacrificed by intracardiac injection of T-61 solution while under 2% isofluorane anaesthetic. In all groups, the operated and nonoperated femora were dissected immediately postsacrifice and then fixed in 10% neutral buffered formalin. All protocols were approved by the St. Michael's Hospital Animal Care Committee.

### 2.2. Cell Isolation, Culture, and Characterization

The cell isolation protocol used in this study was modified from our previous protocol [16] and has been published elsewhere [28]. Briefly, bone marrow was flushed from the medullary canals of rat tibiae and femora with phosphate buffered saline (PBS). Washout solution was collected and subsequently centrifuged at 360*g* for 10 minutes at 18°C. The resulting cell pellet was resuspended in endothelial basal medium (EBM-2) supplemented with EGM-2 MV SingleQuots™ (Lonza, Walkersville, MD). The cell solution was transferred to a T-75 flask previously coated with fibronectin (10 mg/mL). Nonadherent cells were removed after 48 h, and the culture was continued for 7-8 days with medium changes every other day.

Cultured cells were characterized according to their ability to uptake Ac-LDL and bind UEA-1 lectin. Cells loaded onto glass coverslips were incubated with 10 *μ*g/mL of Alexa Fluor® 594 Ac-LDL (Molecular Probes, Eugene, OR) for 4 h at 37°C and 5% CO_2_. Cells were then fixed with 2% paraformaldehyde for 10 minutes, incubated with 20 *μ*g/mL of FITC UEA-1 lectin (Sigma-Aldrich, St. Louis, MO) overnight at 4°C, and subsequently visualized on a fluorescence microscope (Nikon Eclipse E800, Tokyo, Japan). Additionally, a tube formation assay was used to assess the angiogenic potential of the cultured cells. Basement membrane Matrigel (BD Biosciences, Franklin Lakes, NJ) diluted 1 : 2 in EBM-2 medium was loaded into a 12-well plate at 300 *μ*L/well and was allowed to polymerize at 37°C and 5% CO_2_ for 15 minutes. EPCs were added onto the Matrigel at 25,000 cells/cm^2^ and were incubated for 24 h at 37°C and 5% CO_2_. After 24 h, the medium was carefully aspirated, and Calcein AM diluted in EGM-2 MV medium was added to each well at 2 *μ*g/mL. Plates were then imaged at 10× objective using fluorescence microscopy (Zeiss Axio Observer Live Cell, Oberkochen, Germany). Ten z-stacks at 10 *μ*m intervals were acquired with ApoTome and shading correction. Sixteen images in a 4 × 4 layout were captured, and individual stacks and tiles were stitched together to create a composite 3D image using Imaris software (Bitplane, Belfast, UK).

### 2.3. Surgical Procedures

The femoral bone defect surgery has been previously described in an earlier study [16]. Briefly, rats were anaesthetized with 2% isofluorane and given 0.05 mg/kg of buprenorphine analgesic preoperatively via subcutaneous injection. The right leg was shaved and then scrubbed with Betadine solution (povidone-iodine, 10%) and 70% ethanol. All further steps took place under sterile conditions. Using a lateral approach, an incision was made in the skin overlying the femur, and the underlying tissue was dissected to expose the bone surface. Two parallel osteotomies were created in the middle 1/3 of the femoral diaphysis using an oscillating saw with 0.9% saline irrigation. The intervening bone segment was removed, and a 5-hole mini-plate (Synthes, Mississauga, Canada) was fixed to the bone with two proximal and two distal 1.5 mm cortical screws. In the delayed treatment groups, the defect was left empty and a standardized closure was performed. In the acute treatment groups, either EPC-loaded Gelfoam or Gelfoam control was placed in the defect prior to closure. Rats were allowed full weightbearing and cage activity postoperatively and were given 0.05 mg/kg of buprenorphine analgesic every 12 hours for the first 48 hours postsurgery.

During the secondary surgery for the delayed treatment groups, anaesthetic and analgesics were given as described above, and the same surgical approach was utilized to access the femur. Subsequently, the defect was cleared of fibrous tissue, and the bone ends were debrided with a scalpel to induce cortical bleeding. A 20-gauge needle was then used to reestablish the medullary canal, which had typically been covered over by endosteal callus and fibrotic tissue. Then, either EPC-loaded Gelfoam or Gelfoam control was placed in the defect prior to closure.

### 2.4. Radiographic Assessment

All animals assigned to receive delayed treatment or no treatment underwent plain radiographic evaluation after the initial 3-week delay. Defects were scored in a blinded fashion using a radiographic scoring system modified from Atesok et al. [16] (Table 1), and defects with scores of 4 or lower were randomized to receive either EPC-loaded Gelfoam or Gelfoam control or to be left empty with no further surgical intervention. Defects receiving scores of 5 or greater were excluded from further study in order to ensure that the repair of the defect was a result of the intervention and not spontaneous bone repair.

Postintervention, standardized radiographs were taken on a biweekly basis to monitor bone repair. Radiographs taken at the 10-week endpoint were graded using the scoring system outlined in Table 1. Two blinded orthopaedic surgeons graded all radiographs, and the average scores were used for analysis. Additionally, defects were characterized as completely united, incompletely united, or nonunited by a blinded orthopaedic surgeon.

### 2.5. MicroCT Analysis

After specimen harvest and plate removal, the operated femora were placed vertically in a poly-ethyl-imid (PEI) holder (16.4 mm × 75 mm) filled with formalin solution. The samples were scanned at 70 kVp and 114 *μ*A in high resolution (1000 projections per 180°; voxel size = 8 *μ*m) on a MicroCT40 system (Scanco Medical, Basserdorf, Switzerland). The integration time of each projection was 300 ms, and a single scan at each projection was conducted (frame averaging = 1). Prior to analysis, the 2D transverse (x-z) grayscale images (1206–1344 total sections) were reconstructed to x-y cross sections (voxel size = 8 *μ*m; image size = 2048 × 2048 pixels). A rectangular region of interest (ROI) encapsulating the osteotomy site at its widest point was drawn on x-y sections, using the margins of native bone as reference points. For each specimen, the same ROI was applied across all slices with visible cortex, creating a 3D rectangular volume of interest (VOI) (mean volume ± standard error (SE): 72.26 ± 1.430 mm^3^). Threshold for morphometric analysis was held constant across all slices and samples (minimum = 263; maximum = 1000), and the VOIs were analyzed for quantitative bone morphometry. Analysis included the following morphometric parameters: bone volume to total volume fraction (BV/TV (1)), trabecular number (Tb.N^∗^ (1/mm)), trabecular separation (Tb.Sp^∗^ (mm)), and trabecular thickness (Tb.Th^∗^ (mm)).

### 2.6. Biomechanical Analysis

After microCT analysis, the femora were taken for biomechanical testing on an MTS Bionix 858 test system (MTS Systems, Eden Prairie, MN). A 12 in-lb torsional load cell (Futek TFF325, Irvine, CA) was utilized to ensure optimal testing accuracy. To prepare each sample, the proximal and distal epiphyseal-metaphyseal segments of the bones were first potted in polymethyl methacrylate (PMMA) dental cement. The diaphyseal defects were centred between two potting casings using a 20 mm gauge length, and the femoral diaphyses were aligned longitudinally to the axis of the machine. The femora were tested in torsion at a displacement of 1°/second until failure of the bone or a total displacement of 40°. Ultimate torque and torsional stiffness were used for analysis.

### 2.7. Statistical Analysis

All statistical analyses were performed on GraphPad Prism version 6.0 (GraphPad Software, Inc., La Jolla, CA). Analyzed values are expressed as mean ± SE. Data were evaluated using two-way analysis of variance (ANOVA), with Tukey's honest significant difference (HSD) multiple comparisons analysis to identify between-group differences. A *p* value of <0.05 was considered statistically significant.

## 3. Results

### 3.1. Isolation and Characterization of EPCs

**Figure 1:**
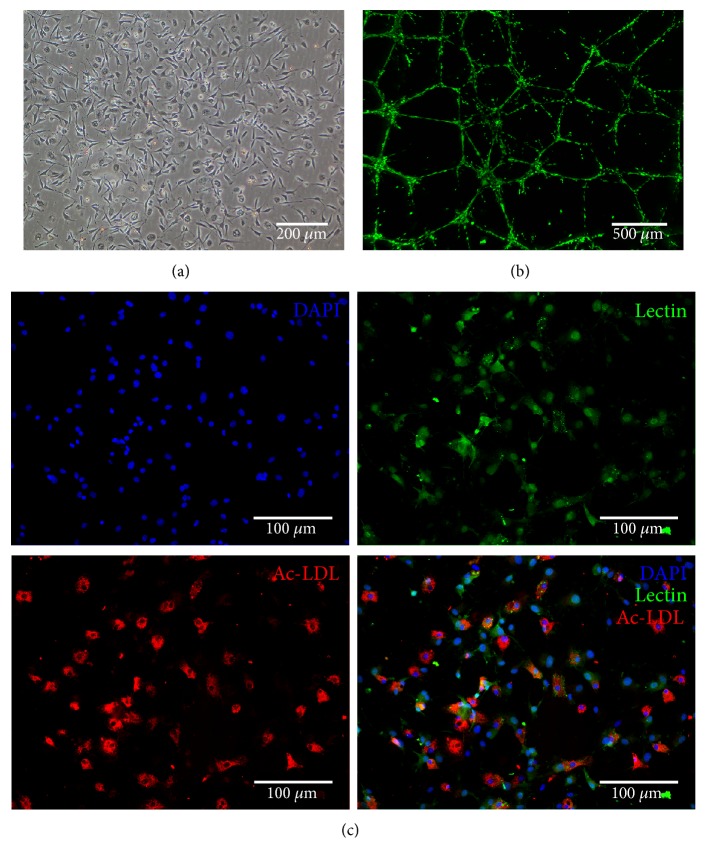
Culture and characterization of rat bone marrow-derived EPCs. (a) Cultured cells displayed spindle-shaped morphology characteristic of EPCs after 7–8 days in culture. (b) EPCs formed tube-like structures after seeding on Matrigel for 24 hours (stained with Calcein AM). (c) EPCs were characterized by Ac-LDL uptake and UEA-1 lectin binding.

The isolated cell population displayed spindle-shaped morphology characteristic of EPCs after 7-8 days in culture (Figure 1(a)). Harvested cells were capable of forming tube-like structures when seeded on Matrigel (Figure 1(b)), and stained double positive for Ac-LDL uptake and UEA-1 lectin binding (Figure 1(c)).

### 3.2. Bone Defect Model

At 3 weeks post bone defect creation, nine animals were excluded from further study as a result of radiographic scores greater than 4 (mean score: 5.11 ± 0.11). Twenty-four animals had scores of 4 or lower, and were thus included for randomization and further study. Prior to repeat intervention, the mean radiographic score of animals in the delayed EPC group (2.00 ± 0.33) was not significantly different from those in the delayed control group (1.75 ± 0.35) or the empty defect control group (1.50 ± 0.42). Histological staining of empty defects 3 weeks post bone defect creation revealed disorganized fibrous tissue and prolapsed muscle tissue filling the bone defect and endosteal callus capping the proximal and distal fracture fragments (data not shown). The mean endpoint radiographic score for the empty defect control group was 2.5 ± 0.62, and six of eight (75.0%) defects went on to radiographic nonunion. The remaining two defects were considered incompletely united; however, only one had bridging bone upon microCT and biomechanical analyses.

### 3.3. Treatment with EPCs Improves Radiographic Healing

Radiographs of animals from the treatment and control groups were analyzed. By two-way ANOVA analysis, animals treated with EPCs had significantly greater radiographic scores at 10 weeks compared to controls (6.89 ± 0.15 versus 4.29 ± 0.67; *p* < 0.001), whereas no difference existed between animals treated in an acute versus delayed fashion. Additionally, all animals receiving EPCs achieved complete union within 10 weeks of treatment irrespective of the timing of cell delivery, whereas control animals demonstrated significantly lower overall union rates.

**Figure 2:**
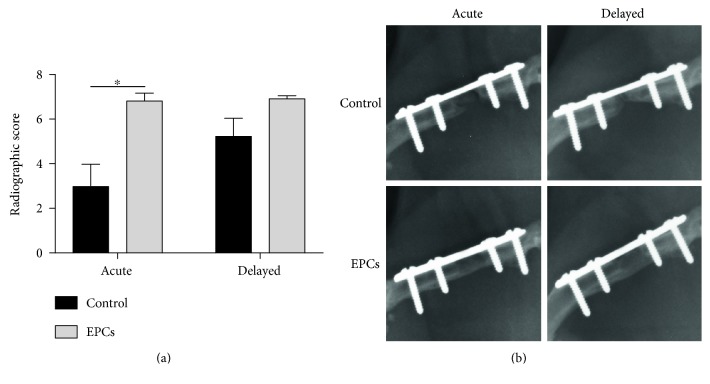
Treatment of bone defects with EPCs improves radiographic healing. (a) Radiographic scores of rat femoral defects at 10 weeks. The difference between EPC treatment and control was statistically significant when treatment was applied acutely but not when treatment was performed in a delayed fashion. No difference was observed between delayed EPC and acute EPC groups. (b) Representative radiographs of defects in each group. Complete radiographic union was observed in all animals receiving EPCs, whereas control groups generally demonstrated incomplete union or nonunion. ∗ denotes significant difference between groups (*p* < 0.05).

In our multiple comparisons analysis, radiographic scores were not significantly different between the delayed EPC and delayed control groups (6.94 ± 0.11 versus 5.25 ± 0.79; *p* = 0.212) (Figure 2(a)). However, all eight (100%) animals in the delayed EPC group achieved complete union, whereas complete union only occurred in three of eight (37.5%) animals in the delayed control group (Table 2). Similarly, all six (100%) animals in the acute EPC group achieved complete union, whereas complete union was not observed in any of the animals in the acute control group. Furthermore, the acute EPC group had significantly higher radiographic scores than the acute control group (6.83 ± 0.33 versus 3.00 ± 0.97; *p* = 0.003). Radiographic scores were not significantly different between delayed and acute application of EPCs, nor between delayed and acute treatment with Gelfoam control. Representative radiographs from each group are shown in Figure 2(b).

### 3.4. Treatment with EPCs Enhances Bone Morphometric Parameters

**Figure 3:**
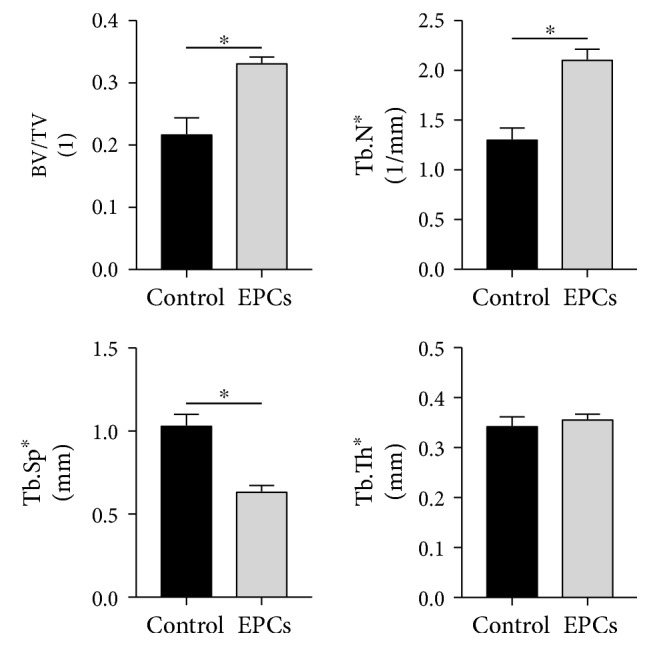
Treatment with EPCs increases bone formation and improves bone morphometric parameters. Global comparison between EPCs and Gelfoam-only controls revealed increased bone volume fraction, increased trabecular number, and decreased trabecular separation when treated with EPCs. Trabecular thickness was unchanged between groups. ∗ denotes significant difference between groups (*p* < 0.05).

Quantitative microCT performed 10 weeks postintervention revealed significant differences in bone morphometry between EPC-treated and control groups. By two-way ANOVA analysis, treatment with EPCs significantly increased bone volume fraction (*p* < 0.001) and trabecular number (*p* < 0.001), and decreased trabecular separation (*p* < 0.001) compared to controls (Figure 3). Trabecular thickness was unchanged between EPC-treated and control groups. No differences were observed between animals treated acutely versus in a delayed fashion.

**Figure 4:**
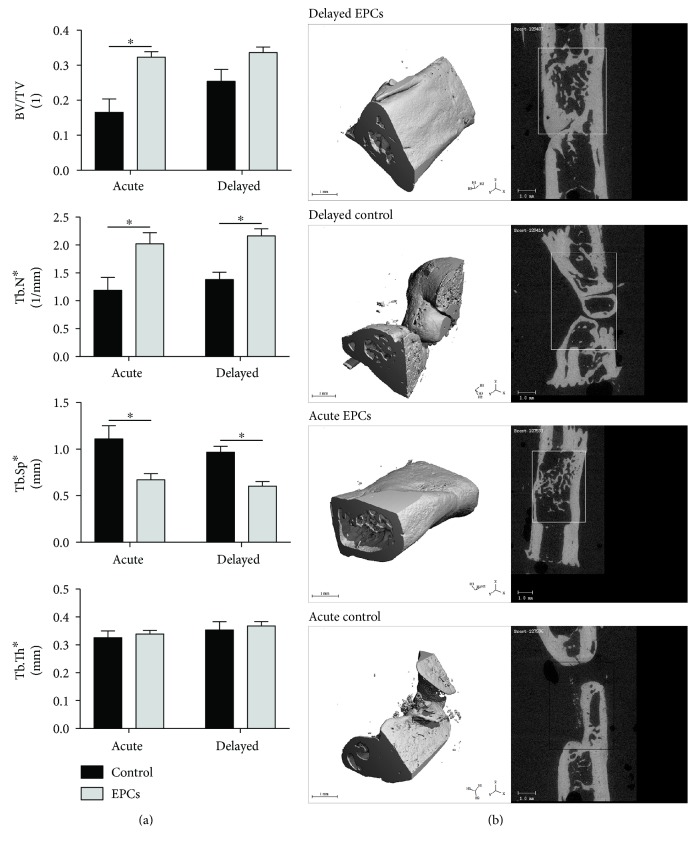
Treatment of bone defects with EPCs enhances bone morphometric parameters. (a) Graphical representation of quantitative microCT parameters. When applied in a delayed fashion, EPCs increased trabecular number and decreased trabecular separation compared to control. When applied acutely, EPCs increased bone volume fraction and trabecular number, and decreased trabecular separation. No differences in any parameter were observed between acute and delayed delivery of EPCs. (b) 3D reconstructions and x-y cross sections of representative defects from each group (BV/TV nearest the group mean). Treatment with EPCs, either in an acute or delayed fashion, demonstrated complete defect bridging at 10 weeks postintervention, whereas incomplete union or nonunion was generally observed in the control groups. ∗ denotes significant difference between groups (*p* < 0.05).

In our multiple comparisons analysis, bone volume fraction was unchanged in the delayed EPC group compared to the delayed control group (*p* = 0.115) (Figure 4(a)). However, compared to the delayed control group, animals treated in a delayed fashion with EPCs demonstrated significantly greater trabecular number (*p* = 0.005) and significantly reduced trabecular separation (*p* = 0.007). When applied in an acute fashion, EPCs significantly increased bone volume fraction (*p* = 0.003), increased trabecular number (*p* = 0.010), and decreased trabecular separation (*p* = 0.005) compared to control. Trabecular thickness was unchanged in all groups. Bone morphometric parameters did not differ between delayed and acute application of EPCs.  Figure 4(b) demonstrates representative 3D reconstructions and x-y cross sections of bone defects from each group.

### 3.5. Treatment with EPCs Increases Biomechanical Strength and Stiffness

Biomechanical testing at 10 weeks postintervention revealed significant improvements in EPC-treated versus control animals. By two-way ANOVA analysis, treatment with EPCs significantly increased ultimate torque (149.53 ± 12.51 versus 45.65 ± 20.44; *p* < 0.001) and torsional stiffness (30.55 ± 1.81 versus 8.34 ± 3.61; *p* < 0.001) compared to controls.

**Figure 5:**
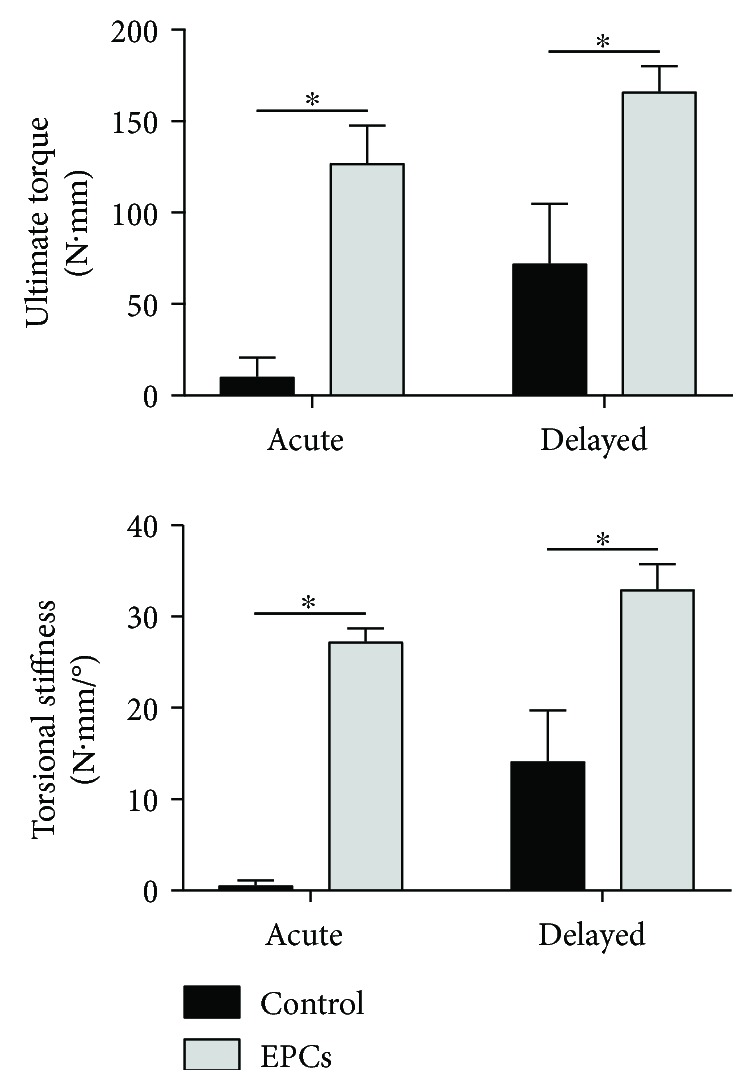
Treatment of bone defects with EPCs increases biomechanical strength and stiffness. Torsional testing revealed that EPC treatment, either in an acute or delayed fashion, increased ultimate torque and torsional stiffness compared to the respective control groups. For both ultimate torque and torsional stiffness, no differences were observed between acute and delayed treatment with EPCs. ∗ denotes significant difference between groups (*p* < 0.05).

In our multiple comparisons analysis, delayed application of EPCs to bone defects significantly improved ultimate torque (166.23 ± 14.05 versus 72.14 ± 32.67; *p* = 0.022) and torsional stiffness (33.01 ± 2.74 versus 14.17 ± 5.56; *p* = 0.003) compared to control (Figure 5). Importantly, the delayed EPC group demonstrated consistent regeneration of functional strength and stiffness, whereas only four of eight (50%) animals in the delayed control group had mechanical stability across the defect. Acute delivery of EPCs resulted in significantly improved ultimate torque (127.26 ± 20.20 versus 10.32 ± 10.32; *p* = 0.012) and stiffness (27.27 ± 1.44 versus 0.56 ± 0.56; *p* < 0.001) compared to acute delivery of Gelfoam-only control. No differences in ultimate torque or torsional stiffness were observed between delayed and acute treatment with EPCs, nor between delayed and acute treatment with Gelfoam control.

## 4. Discussion

The reconstruction of bone defects resulting from trauma or nonunion remains a considerable challenge for orthopaedic surgeons. While acute treatment with EPCs has proven effective in small animal models, patients presenting with traumatic fractures neither have a readily available source of EPCs nor have a biological environment at the fracture site conducive to acute bone reconstruction. In the clinical setting, EPCs would likely be applied during a later secondary surgery, creating a discrepancy between current models and potential clinical use. The present study aimed to address this discrepancy, and our findings indicate that EPCs delivered via open surgery to a previously established bone defect are indeed capable of inducing bone repair. Although the volume of bone formed upon delayed delivery of EPCs did not differ significantly from the control group, the rate of union and quality of the bone were far superior when defects were treated with EPCs. Furthermore, at 10 weeks posttreatment, defects treated in a delayed fashion with EPCs recovered to approximately 65% of nonoperated contralateral bone strength. The mechanical superiority of defects treated with EPCs may in part be attributed to the more abundant and more tightly spaced trabeculae in the EPC group; however, EPCs appeared to contribute significantly by enhancing the rate and quality of defect union. Thus, EPCs administered to nonhealing bone defects evidently enhanced bone healing when compared to control treatment, and therefore, EPCs could be a clinically translatable therapy aimed at the reconstruction of osseous defects in a delayed fashion.

As part of our objective, we aimed to evaluate the influence of timing of EPC application on defect repair. Comparison between acute and delayed EPC treatment demonstrated no significant differences in bone repair between the groups, suggesting that treatment with EPCs at either an acute or delayed stage is effective for the repair of bone defects. These findings are inconsistent with previous investigations of delayed defect reconstruction using cellular and molecular therapies, as delayed treatment has been unable to induce healing responses equivalent to those observed with acute treatment [20, 21]. These differences may be explained by our secondary surgical procedure in which we elevated the soft tissue envelope and reestablished the medullary canal in a comparable fashion to that used clinically in exchange nailing procedures. While exchange nailing stimulates bone union by replacing a smaller unreamed nail with a larger reamed nail to enhance the stability at the fracture site, reaming the medullary canal for placement of the nail stimulates periosteal circulation and likely promotes novel angiogenic stimuli capable of enhancing bone repair [29]. Similarly, in the model used in the current study, elevation of the soft-tissue envelope and opening of the medullary canal may promote sufficient circulation and angiogenic stimuli to induce a healing response in combination with EPCs. Thus, the ability of EPCs to induce adequate bone repair when treated either immediately after fracture or at a delayed time point demonstrates the potential clinical utility of EPCs in bone defect reconstruction.

The mechanisms of EPC-induced bone repair remain incompletely understood. However, it is well established that a complex, coordinated coupling of angiogenesis and osteogenesis is essential to bone regeneration. In normal healing fractures, BMP-2 and BMP-7 are upregulated at 1 and 14–21 days postfracture, namely, the acute inflammatory stage and the stage of endochondral ossification [23]. Conversely, these BMPs are downregulated in models of atrophic nonunion compared to standard healing controls [30, 31]. Increased VEGF expression after EPC application may help to stimulate BMP-2 expression in local endothelial cells [13, 14, 32]. Synergistically, BMP-2 also stimulates VEGF expression in osteoblasts, further suggesting the coupling of angiogenesis and osteogenesis [33]. Thus, EPC-induced expression of VEGF and BMP-2 [13, 14], even after significant delay, may help to overcome the deficient signalling present in fracture nonunion and may initiate the inflammatory and endochondral phases of bone repair through regulation of angiogenesis and osteogenesis. Alternatively, the secondary surgical procedure is likely to cause an inflammatory reaction, which may stimulate EPC activity via IL-1 and IL-6 signalling [24–26]. Still, further investigation into the molecular pathways involved in EPC-mediated bone repair is required, and identification of these mechanisms and their interaction with inflammatory processes may aid in tailoring EPC treatments for clinical use.

The limitations of this study include the investigation of a single delayed time point, and the spontaneous union observed in a small subset of animals. First, the 3-week delay used in this study was chosen based on the accelerated biology observed in rodent fracture repair in comparison to humans [34]. Bone grafting immediately after open fracture is not performed clinically due to risks of infection and graft resorption resulting from the inflammatory reaction [27, 35]. Instead, delayed bone grafting is normally performed once the soft tissue envelope has adequately healed and inflammation has subsided, often between 6 and 8 weeks postinjury [35]. In comparison to high-energy open fractures in human long bones, the rat model utilized in this study achieves soft tissue closure relatively early after the operation because minimal iatrogenic damage occurs during the fracture surgery. Three weeks postfracture, inflammation has significantly decreased [22, 23], re-epithelialization has occurred, and based on histological evidence not included in this study, the defect has been filled with fibrous tissue. All of these factors considered, we concluded that a 3-week delay in our rat model sufficiently replicated the clinical scenario. Still, further investigation with later time points would strengthen the applicability of this study. Second, it could be argued that the regeneration of bone was a result of spontaneous union and not the administration of EPCs. However, animals were only subjected to randomization if their radiographic score at 3 weeks was 4 or lower, so as to include only animals with nonhealing defects. Additionally, we confirmed the nonhealing nature of the bone defects in the study by including a group of animals in which the defect was left empty with no secondary intervention. The failure of defect bridging in this group provides sufficient evidence to conclude that bony regeneration in the treatment groups was indeed a result of secondary surgical intervention and not natural bone repair.

## 5. Conclusion

Our results indicate that EPCs are capable of enhancing radiographic and morphometric bone repair when applied 3 weeks after bony injury, and promote greater and more consistent recovery of biomechanical function compared to a control group. Importantly, the healing response observed upon delayed surgical treatment did not differ from that of acute treatment. These data suggest that open surgical delivery of EPCs on a collagen carrier may be effective when used in a delayed fashion for the treatment of nonhealing bone defects in a clinically relevant scenario. Based on the results from this study, EPC-based therapy could represent a substantial advance for the treatment of nonunions and traumatic bone defects in humans, and further research aimed at bringing this novel therapy to the clinical realm is warranted.

## Conflicts of Interest

The authors declare that there is no conflict of interest regarding the publication of this paper.

## Figures and Tables

**Table 1:** Radiographic scoring system.

Defect filling	Callus density	Score
0%	N/A	0
1–25%	Low	1
	High	2
26–50%	Low	3
	High	4
51–75%	Low	5
	High	6
76–100%	Low	7
	High	8

**Table 2:** Radiographic union rates.

	Acute		Delayed	
	Control	EPCs	Control	EPCs
Complete union	0% (0/6)	100% (6/6)	37.5% (3/8)	100% (8/8)
Incomplete union	50% (3/6)	0% (0/6)	50% (4/8)	0% (0/8)
Nonunion	50% (3/6)	0% (0/6)	12.5% (1/8)	0% (0/8)

EPCs: endothelial progenitor cells.
